# Nationwide pediatric injury surveillance across two successive system disruptions in South Korea, 2016–2024

**DOI:** 10.1186/s40621-026-00708-y

**Published:** 2026-08-14

**Authors:** Sang Il Han, Jeeho Han

**Affiliations:** 1 Department of Emergency Medicine, Uijeongbu Eulji Medical Center, Eulji University, Uijeongbu-si, Republic of Korea; 2 Department of Pediatrics, Asan Medical Center, 88 Olympic-ro 43-gil, Songpa-gu, Seoul, 05505 Republic of Korea

**Keywords:** Pediatric injury, Emergency department surveillance, COVID-19, Healthcare workforce disruption, Interrupted time-series analysis, South Korea

## Abstract

**Background:**

Long-term trends in emergency department (ED)-based pediatric injury surveillance during and after the COVID-19 pandemic remain poorly characterized, particularly in settings that experienced additional system-level disruption. In February 2024, South Korea experienced a second healthcare-system disruption following the mass resignation of trainee physicians from teaching hospitals after a government decision to expand medical school admissions.

**Methods:**

We analyzed National Emergency Department Information System data for children aged under 10 years with injury diagnoses (S00–T79) or external cause codes (V01–Y98), January 2016 to December 2024 (108 months). Monthly ED-recorded injury visit rates were calculated using resident-registration pediatric population denominators. Interrupted time-series analysis used two prespecified breakpoints (COVID-19 onset, March 2020; mass resignation of trainee physicians, February 2024). Recovery Indices compared 2023 with 2018–2019, stratified by resource intensity (KTAS 1–2, ICU, death, or transfer-in) and facility type.

**Results:**

Among 2,783,933 pediatric injury-related ED visits over 9 years, population-adjusted injury contact burden remained incompletely recovered by 2023 (Recovery Index 78.7% after adjusting for a 20.3% pediatric population decline; raw Recovery Index 62.7%), with the transfer-excluded resource-intensity group recovering more completely than the non-resource group (Recovery Index 76.2% vs. 62.3%), suggesting a post-COVID shift in the recorded ED case mix. Regional emergency centers recovered most by 2023 (71.5%) but showed the largest decline at the February 2024 breakpoint (β=−88.7 per 100,000), while non-teaching facilities showed no detectable change (β=+2.4). The level change at the February 2024 breakpoint was − 157.9 per 100,000 (95% CI − 223.7 to − 92.0), against − 183.3 (95% CI − 220.2 to − 146.5) at COVID-19 onset.

**Conclusions:**

ED-recorded pediatric injury contacts in South Korea remained below pre-pandemic levels, while the recorded case mix showed a relative shift toward more resource-intensive presentations. The 2024 trainee-physician workforce disruption was associated with a second, facility-selective decline that was largest in regional emergency centers and not detectable in other emergency facilities; with 11 months of follow-up these estimates are preliminary. These findings show that pediatric injury surveillance during healthcare-system disruption should not rely on visit counts alone; case mix and facility-tier distribution are needed to interpret ED-recorded injury trends.

**Supplementary Information:**

The online version contains supplementary material available at https://doi.org/10.1186/s40621-026-00708-y.

## Background

Pediatric injuries are a leading cause of emergency department (ED) visits and preventable morbidity in children [1]. The COVID-19 pandemic substantially disrupted pediatric ED utilization worldwide, with studies documenting 30–70% decreases in injury-related visits [2–4]. However, most studies examined only the acute pandemic phase [5, 6], leaving the recovery trajectory unknown. Previous Korean studies have examined overall pediatric ED utilization or total visit trends, but injury-specific recovery patterns and facility-level variation remain uncharacterized. Two questions remain unanswered. First, has the composition of pediatric injury ED visits returned to baseline, or has a new equilibrium emerged? If fewer but higher-acuity children now present, the resources required to care for each child may not decline proportionally with volume. Second, does the recovery pattern differ across facility types? Regional emergency centers (typically the facilities with dedicated pediatric emergency capacity) serve a different role from local centers and smaller facilities, and differential recovery across these tiers may change the facility-tier distribution of ED-recorded pediatric injury care.

South Korea offers a well-suited setting for examining these questions. Throughout the pandemic, the country maintained comprehensive national ED surveillance. It also experienced rapid birth-cohort contraction, with the total fertility rate falling from 1.17 to 0.72 over 2016–2023 [7]. A second major system disruption occurred in February 2024, when a government plan to expand medical school admissions by 2,000 places prompted a large-scale resignation of trainee physicians from teaching hospitals [8]. South Korea has approximately 13,000 trainee physicians, concentrated in the roughly 100 teaching hospitals that also operate the country’s higher-level emergency centers, and more than 90% of them had left their posts within weeks [9, 10]. The workforce loss was accompanied by disruption to hospital services [9]; subsequent single-center data from a tertiary teaching hospital showed reduced daily admissions, shorter length of stay, and lower inpatient expenditure after February 2024, consistent with constrained operating capacity during the shortage [10]. Because pediatric emergency care in these institutions depends substantially on trainees, the resulting reduction in service capacity was not distributed evenly across the emergency care system. This sequence allowed us to examine how successive disruptions were associated with children’s injury-related ED use. We aimed to describe 9-year trends in ED-recorded pediatric injury care in South Korea, quantify post-COVID recovery and case-mix change, and examine whether the 2024 trainee-physician workforce disruption was associated with differential changes across facility tiers.

## Methods

### Study design and data source

We conducted a population-based time-series study using the National Emergency Department Information System (NEDIS), which captures approximately 95% of ED visits in South Korea [11]. The study period spanned January 2016 to December 2024 (108 months), bracketing two prespecified system-level events (COVID-19 onset and the February 2024 mass resignation of trainee physicians); the primary interrupted time-series model is specified in the Statistical analysis section.

## Study population

All ED visits by children aged under 10 years with a primary or secondary injury diagnosis (ICD-10: S00–T79) or external cause code (V01–Y98) were included. Procedural complications (T80–T88) and sequelae (T90–T98) were excluded. We restricted the cohort a priori to children aged under 10 years to define a younger-child injury surveillance population in which ED use is strongly dependent on caregiver care-seeking, and to avoid combining these contacts with adolescent injury patterns that may reflect different mechanisms, including intentional self-harm, violence, and independent risk behaviors.

## Variables and definitions

Age was stratified as 0–11 months, 12–23 months, 2–4 years, and 5–9 years. Injury mechanism was classified using the NEDIS injury circumstance variable: falls, traffic, struck/collision, burns, and other/unspecified. Facility type was classified as regional emergency center (level A), local emergency center (level B/C), and other emergency facilities. In South Korea, regional emergency centers are predominantly located within large tertiary hospitals that also serve as teaching institutions with substantial trainee physician staffing; other emergency facilities are typically smaller non-teaching hospitals. Facility-stratified rates used the national pediatric population as denominator. These rates therefore represent population-level contact burden by facility tier, not facility-specific performance. Resource-intensive visits were defined as those meeting any of: Korean Triage and Acuity Scale (KTAS) level 1–2, ICU admission, in-hospital death, or arrival by inter-facility transfer. This prespecified pragmatic composite was intended to capture visits likely to require substantial ED and downstream resources regardless of final disposition. Admission was not included because it may reflect institutional thresholds rather than clinical resource need; the four included criteria each independently signal high resource demand at the point of ED care. Inter-facility transfer-in was retained because it reflects a prior clinical judgment that the child required escalation beyond the capability of the referring facility, signalling resource demand at ED entry even where practice patterns vary.

## Population denominators

Monthly population counts for children aged under 10 years were obtained from the Korean Statistical Information Service (KOSIS) resident registration data by single year of age [12].

### Statistical analysis

Interrupted time-series. Segmented linear regression modeled the population-based rate per 100,000 children. The primary model used the full 108-month study period with two prespecified breakpoints: COVID-19 onset (March 2020) and the mass resignation of trainee physicians (February 2024). Seasonality was adjusted using harmonic terms (sine and cosine, 12-month period). Standard errors used the Newey-West heteroskedasticity and autocorrelation consistent estimator with lag length selected by rule of thumb (lag = 4 for 108 monthly observations). Analyses were performed in Python 3.12.3 using pandas and statsmodels 0.14.6. Segmented models were fitted by ordinary least squares with heteroskedasticity- and autocorrelation-consistent covariance (cov_type = “HAC”, maxlags = 4, Bartlett kernel, without small-sample correction; normal-based inference). Analysis code is available from the corresponding author on request. Facility-type interaction was tested by pooling all facility types in a single model with facility × period interaction terms (cluster-robust standard errors by month). Facility heterogeneity at COVID-19 onset was assessed using a joint Wald test of the facility-by-COVID level and facility-by-post-COVID slope interaction terms; heterogeneity at the February 2024 event was assessed using a joint Wald test of the facility-by-2024 level interaction terms. Sensitivity analyses included shifting the COVID-19 breakpoint by ± 1 and ± 2 months and the February 2024 breakpoint by ± 1 month, to test robustness to exact timing (Supplementary eTable 1), refitting the primary model with eleven monthly indicator variables in place of harmonic terms (Supplementary eTable 7), examining KTAS 1–2 visits alone and reconstructing the resource-intensive composite after excluding inter-facility transfer (Supplementary eTables 5 and 6), and placebo breakpoint tests placed at five time points within the stable pre-pandemic baseline (January 2016 – February 2020) to verify that the ITS did not detect level changes where none was expected (Supplementary eTable 2).

Recovery Index (RI). RI = (2023 monthly average / 2018–2019 monthly average) × 100. An RI of 100% indicates complete return to baseline. We calculated RI for overall volume, population-adjusted rate, resource-intensive visits, facility types, injury mechanisms, and access patterns (ambulance, off-hours).

Compositional change. Proportions of mechanism, acuity, and disposition were compared between 2018 and 2019 and 2022–2023 using independent-samples t-tests. This two-year window was chosen to stabilize monthly proportion estimates and differs from the single-year Recovery period (2023) used in Table 1; the two windows are therefore not directly interchangeable, and reported compositional comparisons in the text refer to the 2018–2019 vs. 2022–2023 contrast. Second disruption analysis. In February 2024, a government announcement to increase medical school admissions by 2,000 places led to a collective resignation of the majority of trainee physicians from South Korean teaching hospitals [8, 9, 13]. We modeled this as a second prespecified breakpoint (February 2024) in a two-breakpoint ITS across the full 108-month study period. Because post-disruption follow-up was limited to 11 months, no separate post-2024 slope-change term was estimated; we therefore report only the immediate level change. Results were stratified by facility type to examine differential disruption impact.

**Table 1:** Characteristics of pediatric injury-related emergency department visits across four study periods, South Korea, 2016–2024

Characteristic	Pre-COVID (2018–2019)	Pandemic (Mar 2020–Dec 2022)	Recovery (2023)	2024 Crisis (Feb–Dec 2024)	*P*-value
Duration (months)	24	34	12	11	*—*
Total injury-related visits, n	761,770	725,658	238,711	156,069	*—*
Monthly visits, mean (SD)	31,740 (4,573)	21,343 (3,738)	19,893 (2,492)	14,188 (2,148)	*< 0.001*
Rate per 100,000/month, mean (SD)	739.3 (106.3)	557.1 (94.6)	581.5 (73.1)	440.8 (62.1)	*< 0.001*
**Sex**,** %**					
Male	59.29	59.33	59.46	59.15	*0.242*
**Age group**,** %**					
0–11 months	6.87	7.30	8.48	8.01	*< 0.001*
12–23 months	16.34	15.35	15.09	13.81	*< 0.001*
2–4 years	28.88	27.18	25.35	24.66	*< 0.001*
5–9 years	47.91	50.18	51.09	53.52	*< 0.001*
**Injury mechanism**,** %**					
Falls	22.66	21.86	22.43	19.25	*< 0.001*
Traffic	23.33	26.15	27.32	25.70	*< 0.001*
Struck/collision	6.27	6.26	5.88	4.69	*< 0.001*
Burns	3.01	2.51	2.39	2.19	*< 0.001*
Other/unspecified	44.74	43.23	41.98	48.17	*< 0.001*
**Acuity & resource use**,** %**					
KTAS 1–2 (high acuity)	0.97	0.97	1.21	1.51	*< 0.001*
Admitted to hospital	2.60	2.56	2.29	2.59	*< 0.001*
ICU admission	0.16	0.17	0.16	0.18	*0.037*
In-hospital death	0.02	0.03	0.02	0.02	*0.326*
Inter-facility transfer in	3.38	3.90	3.73	3.31	*< 0.001*
Resource-intensive (composite)*	4.30	4.82	4.89	4.69	*< 0.001*
**Access / arrival**,** %**					
Ambulance arrival	6.94	8.00	8.73	9.37	*< 0.001*
Off-hours presentation	56.21	56.57	55.51	56.41	*< 0.001*
**Facility type**,** %**					
Regional emergency centers (Level A)	27.94	27.93	31.87	23.87	*< 0.001*
Local emergency centers (Level B/C)	49.46	50.56	49.02	49.38	*< 0.001*
Other emergency facilities	22.60	21.51	19.11	26.74	*< 0.001*

KTAS, Korean Triage and Acuity Scale; SD, standard deviation

*Resource-intensive visits were prespecified as a pragmatic composite of KTAS level 1–2, ICU admission, in-hospital death, or inter-facility transfer in. Percentages were computed from individual-level records. Admission was not included because it may reflect institutional thresholds rather than clinical resource need

**Fig. 1:**
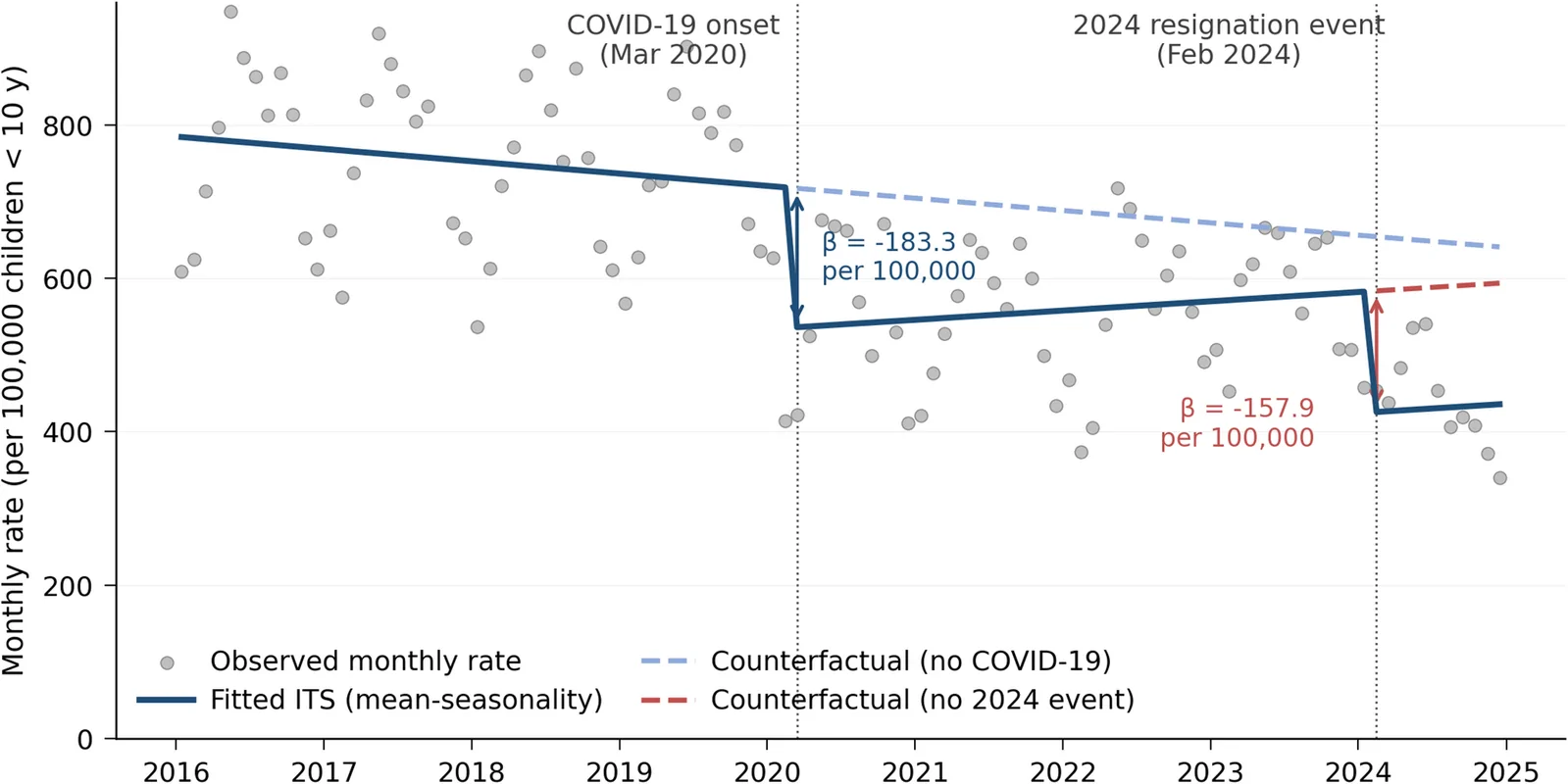
Monthly population-based rate of pediatric injury-related ED visits, South Korea, January 2016 to December 2024, with a two-breakpoint interrupted time-series model. Grey circles show observed monthly rates per 100,000 children aged under 10 years. The solid line shows the fitted interrupted time-series model, averaged across months to align with the observed data. Two prespecified breakpoints are shown: COVID-19 onset (March 2020) and the February 2024 mass resignation of trainee physicians. Dashed lines show counterfactual trajectories. COVID-19 onset was associated with an immediate level change of − 183.3 per 100,000 children (95% CI − 220.2 to − 146.5), followed by a recovery slope of + 2.35 per 100,000 per month (95% CI 0.80 to 3.89). The February 2024 event was associated with a further level change of − 157.9 per 100,000 children (95% CI − 223.7 to − 92.0). Because post-disruption follow-up was limited to 11 months, the post-2024 slope was not estimated

**Fig. 2:**
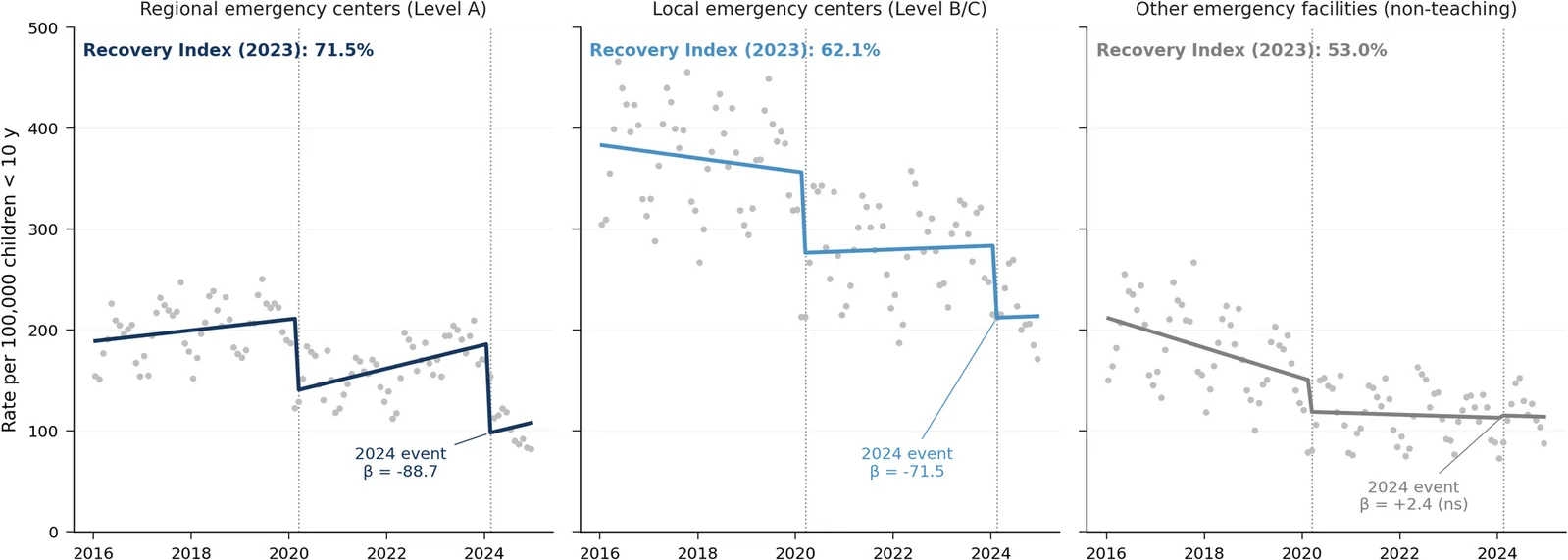
Facility-stratified interrupted time-series of pediatric injury-related ED visit rates across three facility tiers, South Korea, 2016 to 2024. Monthly rates per 100,000 children aged under 10 years are shown separately for regional emergency centers (Level A), local emergency centers (Level B/C), and other emergency facilities (non-teaching). Rates were calculated using the total national pediatric population as the denominator and therefore represent population-level contact burden by facility tier. All three facility types showed significant declines at COVID-19 onset. By 2023, Recovery Indices were 71.5% for regional centers, 62.1% for local centers, and 53.0% for other facilities. At the February 2024 event, regional centers showed the largest level decline (β = −88.7 per 100,000; p < 0.001), local centers a smaller decline (β = −71.5; p < 0.001), and non-teaching facilities no detectable change (β = +2.4; p = 0.769). In the pooled interaction model, facility heterogeneity was significant both at COVID-19 onset (facility × COVID interaction, level and slope terms jointly, p < 0.001) and at the February 2024 event (facility × 2024 level interaction, p < 0.001).

P-values compare across the four periods using one-way analysis of variance (monthly-level counts and rates) and chi-square tests on pooled counts (percentages). The 27 months not assigned to any of the four periods (Jan 2016 – Dec 2017, Jan–Feb 2020, and Jan 2024) were excluded from Table 1 comparisons but retained in the interrupted time-series analysis (Table 2, Figures 1–2). Percentages in Table 1 are pooled over all visits within each period; the compositional t-tests reported in the main text compare monthly-level proportions between 2018–2019 and 2022–2023 and therefore yield values that differ from the Table 1 percentages by up to approximately 0.1 percentage point for the same variable and period

Prespecified subgroup analyses. We prespecified stratification by metropolitan area (capital region vs. non-capital), insurance type (National Health Insurance vs. Medical Aid), and facility type to examine whether recovery and disruption impacts were geographically, socioeconomically, or structurally patterned.

### Reporting guideline

This study is reported with reference to the STROBE Statement.

### Ethics approval

This study was approved by the Institutional Review Board of Asan Medical Center, Seoul, Republic of Korea (approval number: 2026 − 0171). The requirement for informed consent was waived because of the retrospective use of de-identified administrative data from the National Emergency Department Information System.

## Results

### Study population

Over 108 months, 2,783,933 injury-related ED visits by children aged under 10 years were identified, including 2,612,700 in the Recovery Index analysis period (2016–2023). The largest age group was 5–9 years (48.5%), followed by 2–4 years (27.7%). Resource-intensive visits accounted for 4.3% of all visits in the pre-COVID period.

### Volume trends and ITS

Annual visits declined from 421,745 in 2016 to 238,711 in 2023 (Table 1). COVID-19 onset was associated with an immediate rate decrease of 183 per 100,000 (95% CI − 220.2 to − 146.5), followed by a recovery slope of + 2.35 per 100,000/month (95% CI 0.80 to 3.89); however, the observed rate had not returned to the pre-COVID baseline by December 2023 (Fig. 1).

### Recovery Index: overall and population-adjusted

By 2023, the raw visit RI was 62.7% (Table 2). The population of children aged under 10 years declined by 20.3% over the study period; after adjustment, the population-based rate RI was 78.7%. The 20.3% population decline did not fully account for the 37.3% visit shortfall, suggesting that factors beyond demographic change contributed to incomplete recovery.

**Table 2:** Recovery Indices and interrupted time-series coefficients for the COVID-19 onset and the 2024 mass resignation of trainee physicians

Subgroup	Recovery Index 2023 (%)	COVID-19 onset (Mar 2020) level β (95% CI, per 100,000)	2024 workforce crisis (Feb 2024) level β (95% CI, per 100,000)
**Overall (primary outcome)**			
All injury visits (count-based RI; rate-based ITS)	62.7	−183.3 (−220.2 to −146.5)^†^	− 157.9 (−223.7 to −92.0)^†^
Population-adjusted rate^‡^	78.7	—	—
**Composition**			
Resource-intensive visits (composite)	71.3	−5.1 (−7.3 to −3.0)^†^	−8.7 (−11.8 to −5.6)^†^
Non-resource visits	62.3	−178.2 (−213.4 to −143.0)^†^	−149.2 (−212.8 to −85.6)^†^
Ambulance arrivals	78.8	−13.2 (−15.7 to −10.7)^†^	−13.9 (−19.0 to −8.8)^†^
High-acuity (KTAS 1–2)	78.1	−1.1 (−1.9 to −0.3) (*p* = 0.010)	−0.7 (−1.9 to 0.5) (*p* = 0.248)
**Facility type**			
Regional emergency centers (Level A)	71.5	−71.6 (−87.1 to −56.1)^†^	−88.7 (−114.3 to −63.1)^†^
Local emergency centers (Level B/C)	62.1	−80.1 (−97.6 to −62.5)^†^	−71.5 (−100.0 to −43.1)^†^
Other emergency facilities	53.0	−31.7 (−41.2 to −22.2)^†^	+ 2.4 (−13.4 to 18.1) (*p* = 0.769)
**Region**			
Capital area (Seoul/Incheon/Gyeonggi)	61.9	−115.0 (−138.5 to −91.5)^†^	−107.8 (−144.7 to −71.0)^†^
Non-capital	63.5	−68.3 (−84.4 to −52.2)^†^	−50.1 (−79.8 to −20.3)^†^

Recovery Index (RI) = (mean monthly value in 2023) / (mean monthly value in 2018–2019) × 100

An RI of 100% indicates complete return to baseline. β coefficients represent immediate level changes in the monthly rate per 100,000 children aged under 10 years, estimated from segmented linear regression on the full 108-month study period with two prespecified breakpoints (March 2020 and February 2024), harmonic terms (sine and cosine, 12-month period) for seasonal adjustment, and Newey-West heteroskedasticity- and autocorrelation-consistent standard errors (lag = 4). Because post-disruption follow-up was limited to 11 months, the post-2024 slope was not estimated. Facility-stratified and region-stratified rates use the national pediatric denominator and therefore represent population-level contact burden by facility tier or region, not facility-specific performance

^†^*p* < 0.001

^‡^Population-adjusted rate uses monthly resident registration denominators for children aged under 10 years (Korean Statistical Information Service); β coefficients are reported on the population-based rate scale and are therefore already adjusted for the 20.3% decline in the pediatric population

### Resource-intensive subgroup

Resource-intensive visits showed a higher Recovery Index (RI 71.3%) than non-resource visits (62.3%). Ambulance arrivals showed the highest recovery (RI 78.8%). The resource-intensive proportion increased from 4.29% in 2018–2019 to 4.80% in 2022–2023 (+ 0.51% points; *p* < 0.001), indicating a shift in the ED-recorded case mix toward more resource-intensive presentations. In ITS analysis, the resource-intensive rate showed a significant recovery slope (β = +0.132 per 100,000/month; *p* = 0.003). In a sensitivity analysis using KTAS 1–2 visits alone, the 2023 Recovery Index was 78.1% and the COVID-19 level change was significant (β = −1.06 per 100,000; 95% CI − 1.87 to − 0.25), while the February 2024 level change was not (β = −0.72; 95% CI − 1.94 to 0.50) (Supplementary eTable 5). Because 75.3% of composite-qualifying visits in the pre-COVID period qualified solely through inter-facility transfer, we also repeated the analysis with transfer excluded (Supplementary eTable 6). The 2023 Recovery Index for this restricted composite was 76.2%, against 62.3% for non-resource visits as originally defined (visits qualifying solely through inter-facility transfer are excluded from both groups), and the COVID-19 level change remained significant (β = −1.23 per 100,000; 95% CI − 2.06 to − 0.41); the February 2024 level change was not significant (β = −0.81; 95% CI − 2.04 to 0.41), while the corresponding inter-facility transfer series showed a level change of − 7.8 per 100,000 (95% CI − 10.5 to − 5.2).

### Facility-type stratification

Recovery patterns differed significantly across facility types (facility × COVID interaction *p* < 0.001 for the level and slope terms jointly; Fig. 2). In stratified ITS, all three facility types showed significant COVID-associated rate declines, though with differing magnitudes and recovery trajectories: local centers (β=−80.1 per 100,000; *p* < 0.001), regional centers (β=−71.6; *p* < 0.001), and other facilities (β=−31.7; *p* < 0.001). Regional centers had been on a rising pre-COVID trajectory, while other facilities declined from an already falling baseline. By 2023, regional centers had the highest RI (71.5%), followed by local centers (62.1%) and other facilities (53.0%). Resource-intensive visits at regional centers recovered to 75.3%, compared with 65.1% at local centers.

### Second disruption: 2024 mass resignation of trainee physicians

In a two-breakpoint ITS model (108 months), the immediate level change at the February 2024 breakpoint was − 157.9 per 100,000 (95% CI − 223.7 to − 92.0; *p* < 0.001), against − 183.3 per 100,000 at COVID-19 onset. The two estimates were not formally compared. This second disruption was facility-selective. Regional emergency centers showed the largest disruption-associated decline (β=−88.7; *p* < 0.001), local centers a smaller decline (β=−71.5; *p* < 0.001), and non-teaching facilities no significant change (β=+2.4; *p* = 0.769) (Table 2; Fig. 2). Facility-specific heterogeneity in the 2024 disruption effect was confirmed in the pooled interaction model (facility × crisis interaction *p* < 0.001). The facilities that showed the most complete recovery from COVID-19 showed the largest decline during the resignation event. With 11 months of follow-up, these estimates are preliminary and characterize the immediate level change only.

A confirmatory analysis stratified by treatment-facility region (Seoul/Incheon/Gyeonggi versus the rest of the country, using the national pediatric denominator throughout) showed a parallel pattern: the immediate level decline at the February 2024 breakpoint was larger in the capital area (β=−107.8 per 100,000; 95% CI − 144.7 to − 71.0) than in non-capital institutions (β=−50.1; 95% CI − 79.8 to − 20.3), with a significant region × crisis interaction (*p* < 0.001).

### Compositional shift

The proportion of traffic-related injuries increased from 23.2% to 27.0% (*p* < 0.001), reflecting an accelerating secular trend (ITS: β = +0.13% points/month; *p* < 0.001) rather than an acute pandemic-associated shift (level change *p* = 0.73). Mechanism-stratified interrupted time-series results for all five injury mechanisms are presented in Supplementary eTable 4. The admission rate decreased from 2.57% in 2018–2019 to 2.27% in 2022–2023 (*p* < 0.001), while the KTAS 1–2 proportion increased from 0.97% to 1.07% (*p* = 0.048). During the 2024 period, the proportion of visits without a specified injury mechanism rose from 41.98% to 48.17%, with a corresponding decline across all specified categories (falls 22.43% to 19.25%; traffic 27.32% to 25.70%; struck or collision 5.88% to 4.69%; burns 2.39% to 2.19%). Month-to-month variability in the unspecified proportion was also greater than in 2023 (standard deviation 2.62 versus 1.49% points). ICD-10 external cause codes were recorded for only 2.3% of visits overall, declining from 2.4% in 2016 to 1.5% in 2024, and could not be used to reclassify mechanism. This pattern may reflect reduced documentation specificity during the workforce disruption, although changes in coding practice cannot be distinguished from changes in case composition; we therefore avoid mechanism-specific interpretation of the 2024 data.

## Discussion

In South Korea, pediatric injury-related ED contact remained below pre-pandemic levels even after accounting for a 20.3% decline in the child population. By 2023, the population-adjusted Recovery Index was 78.7%, indicating that demographic contraction alone did not explain the persistent shortfall. These ED-based estimates cannot distinguish changes in injury occurrence from changes in care-seeking, access, or service capacity. A second change was observed in February 2024, when ED-recorded injury contact declined selectively across facility tiers at the time of the mass resignation of trainee physicians. The level change was of similar order to that at COVID-19 onset (β=−158 versus β=−183 per 100,000; the two estimates were not formally compared), indicating that ED-recorded pediatric injury care had not returned to a stable pre-pandemic pattern before the second disruption [14].

The 2024 decline was concentrated in higher-level emergency facilities. Regional emergency centers, which had the highest Recovery Index by 2023 (71.5%), showed the largest decline at the February 2024 breakpoint (β=−88.7 per 100,000; *p* < 0.001), whereas other emergency facilities showed no detectable change (β=+2.4; *p* = 0.769); facility heterogeneity was confirmed by a joint interaction test in the pooled model (*p* < 0.001). With only 11 months of follow-up, these estimates characterize the immediate level change rather than a sustained post-disruption trend, and whether the decline persisted, partially resolved, or deepened cannot be determined. During the disruption, government and emergency medical services measures increasingly prioritized acuity-based distribution of patients, which may have redirected lower-acuity transports away from tertiary centers [15]. Greater reliance on trainee physicians at regional centers is another plausible contributor, although staffing structure was not directly measured; we could not distinguish redirection of transports from changes in caregiver care-seeking. We therefore interpret the facility-selective pattern as evidence that ED-based surveillance is shaped by how the emergency care system operates, rather than as a direct measure of injury occurrence.

Case mix also differed during recovery. Resource-intensive visits recovered more completely than non-resource visits, and this finding persisted after excluding inter-facility transfer: the restricted composite reached 76.2% of baseline against 62.3% for non-resource visits, and KTAS 1–2 visits showed a similar pattern (Recovery Index 78.1%). By contrast, the apparent 2024 decline in the composite as originally specified was no longer detected once transfer was excluded, while the transfer series itself declined sharply. The original composite finding therefore primarily reflects changes in transfer patterns rather than independently measured clinical acuity, and should not be interpreted as evidence of a change in clinical severity. Reduced receiving capacity at teaching hospitals is a plausible explanation, although capacity was not measured directly. These findings are consistent with multicenter pediatric ED data showing greater relative concentration of higher-severity or higher-resource injury presentations despite lower overall volumes during the pandemic [16]. They extend prior Korean NEDIS studies [17, 18] by adding population adjustment, resource-intensity and facility-tier analyses, and follow-up through a second system disruption.

Subgroup analyses suggested demographic, geographic and socioeconomic heterogeneity, but should remain hypothesis-generating. Children aged 0–11 months were the only age group whose population-adjusted Recovery Index reached or exceeded 100% by 2023 (104.3%; Supplementary eTable 3), indicating that infant injury-related ED contact did not contract in proportion to the pediatric population. The February 2024 level decline was larger in capital-area than in non-capital institutions (β=−108 versus − 50 per 100,000; interaction *p* < 0.001), and medical-aid beneficiaries showed lower recovery than national health insurance beneficiaries (Recovery Index 52.4% versus 63.9%; interaction *p* < 0.001), consistent with broader evidence that social determinants shape pediatric emergency care-seeking and outcomes [19]. Recorded in-hospital death, by contrast, remained rare and stable across the study period (0.019% to 0.026% of visits; *p* = 0.33), and the February 2024 level change was not statistically significant (β = −0.041 per 100,000; 95% CI − 0.084 to 0.002). These data do not support inference about care quality in either direction. Only 30 deaths were recorded during the 2024 period, and NEDIS captures deaths recorded by the reporting institution, in the emergency department or after admission, but not deaths occurring after transfer elsewhere.

A decline in ED-recorded contact should not be interpreted as a decline in the need for emergency care. Because injury occurrence is not observed, lower contact may reflect reduced service capacity, redirected transports, altered care-seeking thresholds, or a combination of these. The facility-selective 2024 pattern is more consistent with a change in where care was delivered and recorded than with a uniform reduction in need, although our data cannot distinguish these mechanisms. Surveillance during system disruption should therefore be interpreted using case mix and facility-tier distribution alongside visit counts, and where recorded contact falls sharply and selectively during a period of reduced capacity, the possibility of unmet need is better assessed through data sources outside the emergency department than inferred from ED counts alone. This is consistent with broader literature linking pediatric ED readiness to patient outcomes [20] and documenting challenges to readiness during system stress [21, 22]. Workforce disruption is also not unique to South Korea: junior doctor industrial action in the United Kingdom [23] and post-pandemic staffing strain in the United States [24] have raised similar concerns about the resilience of emergency services. Nationwide analyses have reported reductions in overall emergency department visits following the February 2024 walkout [13], and the pattern described here should be read as the pediatric injury component of that broader change rather than as an injury-specific effect.

This study has several strengths, including nationwide coverage of approximately 95% of ED visits, a 9-year observation period spanning two system disruptions, monthly resident-registration denominators, and analyses stratified by resource intensity and facility tier. Several limitations warrant emphasis. First, NEDIS cannot distinguish changes in injury occurrence from changes in care-seeking, access, or service capacity. Second, the resource-intensive composite is pragmatic and has not been externally validated, and is heavily influenced by inter-facility transfer, which responds to system capacity as well as to clinical need; the transfer-excluded analyses should therefore guide interpretation of the 2024 period. Third, our data comprise injury-related visits only, because non-injury pediatric ED visits were not available under the approved data extract, so the 2024 decline cannot be considered injury-specific. Fourth, only 11 months followed the 2024 breakpoint, and facility-level interpretation is ecological because staffing structure, receiving capacity, transport redirection, and caregiver behavior were not directly measured. Fifth, interrupted time-series estimates represent associations with event timing rather than causal effects, and the 2024 physician workforce disruption affected all medical specialties. Mechanism classification may also have been affected by documentation quality, and ICD-10 external cause codes were recorded for only 2.3% of visits and could not be used for reclassification. Deaths recorded by the reporting institution do not capture deaths occurring after transfer to another institution and therefore do not represent complete injury case fatality. Facility-stratified rates represent population-level contact burden by facility tier rather than facility-specific performance, and medical-aid subgroup analyses are constrained by small absolute numbers. Future studies linking individual-level workforce, transport, socioeconomic, and post-transfer outcome data could better distinguish the mechanisms underlying these patterns.

## Conclusions

In this nationwide ED-based surveillance study, pediatric injury-related ED contacts in South Korea remained below pre-pandemic levels, while the recorded case mix showed a relative shift toward more resource-intensive presentations. The 2024 trainee-physician workforce disruption was associated with a second, facility-selective decline that was largest in regional emergency centers and not detectable in other emergency facilities; with 11 months of follow-up these estimates are preliminary. These findings show that pediatric injury surveillance during healthcare-system disruption should not rely on visit counts alone; case mix and facility-tier distribution are needed to interpret ED-recorded injury trends.

## Supplementary Information

Supplementary Material 1

## Data Availability

The data used in this study were obtained from the National Emergency Department Information System (NEDIS) under a data use agreement with the National Emergency Medical Center of Korea. The data are not publicly available due to privacy restrictions. Statistical code is available from the corresponding author upon reasonable request.
