# Supplementary material for: Nationwide pediatric injury surveillance across two successive system disruptions in South Korea, 2016–2024
Source: Inj Epidemiol. 2026 Aug 14;13:73. doi: 10.1186/s40621-026-00708-y (PMC13753310; doi:10.1186/s40621-026-00708-y)
Supplement: Supplementary file 1 [file 40621_2026_708_MOESM1_ESM.docx]

**Supplementary material**

*Nationwide Pediatric Injury Surveillance Across Two Successive System Disruptions in South Korea, 2016–2024*

Unless otherwise stated, tables in this supplement use the same model specification as Table 2 and Figures 1–2 of the main text. eTable 1 varies the breakpoint timing, eTable 2 uses a single-breakpoint model restricted to the pre-pandemic period, and eTable 7 refits the primary model under an alternative seasonality specification, as described in each table.

**eTable 1. Breakpoint shift sensitivity for the overall monthly rate**

The COVID-19 breakpoint was shifted by ±1 and ±2 months, and the February 2024 breakpoint by ±1 month.

| **Scenario** | **COVID-19 level β (95% CI)** | **February 2024 level β (95% CI)** |
| --- | --- | --- |
| Main (Mar 2020 / Feb 2024) | −183.3 (−220.2 to −146.5)† | −157.9 (−223.7 to −92.0)† |
| COVID −1 month (Feb 2020) | −206.7 (−235.6 to −177.8)† | −160.0 (−223.5 to −96.4)† |
| COVID +1 month (Apr 2020) | −155.2 (−222.6 to −87.8)† | −152.9 (−220.6 to −85.3)† |
| 2024 event −1 month (Jan 2024) | −181.0 (−218.3 to −143.7)† | −144.0 (−216.6 to −71.5)† |
| 2024 event +1 month (Mar 2024) | −183.7 (−219.8 to −147.6)† | −169.3 (−227.8 to −110.8)† |
| COVID −2 months (Jan 2020) | −191.8 (−222.2 to −161.4)† | −148.5 (−209.8 to −87.1)† |
| COVID +2 months (May 2020) | −136.1 (−220.9 to −51.3), p=0.002 | −150.4 (−219.8 to −80.9)† |

The COVID-19 breakpoint was shifted by ±1 and ±2 months because pandemic-related behavioral change and healthcare disruption are likely to have evolved over a broader window in monthly aggregated data; the February 2024 event was shifted by ±1 month only, as it was a more discrete event with a substantially shorter post-event series. β coefficients are immediate level changes in the monthly rate per 100,000 children aged under 10 years. All models use the primary specification: segmented linear regression on the monthly population-based rate with harmonic terms (sine and cosine, 12-month period) for seasonality and Newey-West heteroskedasticity- and autocorrelation-consistent standard errors (lag = 4). †p < 0.001. The direction and approximate magnitude of both level changes are preserved across all shifts.

**eTable 2. Placebo breakpoint tests in the pre-COVID period**

False breakpoints were placed at five time points within the stable pre-pandemic baseline (January 2016 – February 2020) to test whether the model detects level changes where none is expected.

| **Placebo breakpoint** | **Level change β (95% CI)** | **P-value** |
| --- | --- | --- |
| Oct 2017 | +34.1 (−44.9 to +113.1) | 0.397 (ns) |
| Jul 2018 | −34.1 (−89.9 to +21.7) | 0.231 (ns) |
| Oct 2018 | +8.7 (−41.5 to +58.8) | 0.734 (ns) |
| Jan 2019 | +20.6 (−40.1 to +81.4) | 0.505 (ns) |
| Apr 2019 | +27.8 (−44.4 to +100.0) | 0.450 (ns) |

Each placebo model is a single-breakpoint interrupted time series restricted to January 2016 through February 2020, so that the actual COVID-19 onset cannot contaminate the estimate. None of the five placebo breakpoints produced a statistically significant level change (all p ≥ 0.23), indicating that the segmented specification did not routinely identify spurious level changes during the stable pre-pandemic period. ns = not statistically significant.

**eTable 3. Age-stratified interrupted time-series analysis**

Rates are age-specific: the numerator is age-specific injury visits and the denominator the corresponding age-specific resident-registration population.

| **Age group** | **RI 2023 (%)** | **COVID-19 level β (95% CI)** | **Recovery slope β** | **February 2024 level β (95% CI)** |
| --- | --- | --- | --- | --- |
| 0–11 months | 104.3 | −167.8 (−197.4 to −138.2)† | +2.76 | −248.3 (−328.1 to −168.6)† |
| 12–23 months | 81.4 | −273.4 (−323.1 to −223.7)† | +1.69 (ns) | −346.5 (−470.1 to −222.8)† |
| 2–4 years | 79.9 | −151.0 (−186.2 to −115.7)† | +1.16 (ns) | −150.7 (−212.0 to −89.4)† |
| 5–9 years | 76.2 | −184.1 (−229.2 to −139.0)† | +2.61 | −132.3 (−199.0 to −65.6)† |

β coefficients are in units of rate per 100,000 children in the specified age band. All models use the primary specification: segmented linear regression on the monthly population-based rate with harmonic terms (sine and cosine, 12-month period) for seasonality and Newey-West heteroskedasticity- and autocorrelation-consistent standard errors (lag = 4). †p < 0.001. Recovery Indices are computed from age-specific rates. Children aged 0–11 months were the only stratum whose population-adjusted Recovery Index reached or exceeded 100% (104.3%). All four strata showed a significant further level decrease at the February 2024 event. ns = not statistically significant.

**eTable 4. Mechanism-stratified interrupted time-series analysis**

Rates use the national pediatric denominator and therefore represent population-level contact burden for each injury mechanism.

| **Mechanism** | **RI 2023 (%)** | **COVID-19 level β (95% CI)** | **Recovery slope β** | **February 2024 level β (95% CI)** |
| --- | --- | --- | --- | --- |
| Falls | 62.1 | −50.9 (−59.8 to −41.9)† | +0.427 | −50.7 (−67.1 to −34.2)† |
| Traffic | 73.4 | −45.9 (−58.7 to −33.1)† | -0.078 (ns) | −55.1 (−73.6 to −36.6)† |
| Struck/collision | 58.8 | −8.1 (−11.1 to −5.2)† | +0.107 | −12.6 (−17.0 to −8.2)† |
| Burns | 49.8 | −5.6 (−6.9 to −4.2)† | +0.101 | −3.2 (−5.1 to −1.3)† |
| Other/unspecified | 58.8 | −72.9 (−88.8 to −56.9)† | +1.789 | −36.3 (−62.4 to −10.3), p=0.006 |

β coefficients are in units of rate per 100,000 children aged under 10 years. All models use the primary specification: segmented linear regression on the monthly population-based rate with harmonic terms (sine and cosine, 12-month period) for seasonality and Newey-West heteroskedasticity- and autocorrelation-consistent standard errors (lag = 4). †p < 0.001. Burn-related injuries showed the lowest 2023 Recovery Index (49.8%) and traffic-related injuries the highest (73.4%). All five mechanisms showed a significant further level decrease at the February 2024 event. Mechanism categories derive from the NEDIS injury circumstance field; the large "other or unspecified" residual class is a known limitation of that field and is discussed in the main text. ns = not statistically significant.

**eTable 5. Interrupted time-series results for KTAS 1–2 visits alone**

Sensitivity analysis using an independent, externally standardized acuity measure in place of the resource-intensive composite. The outcome is the monthly population-based rate of KTAS 1–2 visits per 100,000 children aged under 10 years.

| **Parameter** | **Estimate** | **95% CI** | **p** |
| --- | --- | --- | --- |
| Recovery Index, 2023 | 78.1% | — | — |
| COVID-19 onset, level change | −1.1 | −1.9 to −0.3 | 0.010 |
| Post-COVID recovery slope | +0.157 | 0.110 to 0.204 | <0.001 |
| February 2024, level change | −0.7 | −1.9 to 0.5 | 0.248 |

The 2023 Recovery Index for non-resource visits was 62.3% for comparison. All models use the primary specification: segmented linear regression on the monthly population-based rate with harmonic terms (sine and cosine, 12-month period) for seasonality and Newey-West heteroskedasticity- and autocorrelation-consistent standard errors (lag = 4). Because post-disruption follow-up was limited to 11 months, the post-2024 slope was not estimated.

**eTable 6. Resource-intensive composite, with and without inter-facility transfer**

The composite as originally specified comprises KTAS 1–2, ICU admission, in-hospital death, or arrival by inter-facility transfer. Because transfer accounts for the majority of qualifying visits, the composite was rebuilt from individual records excluding transfer, and each component was examined separately.

| **Outcome** | **RI 2023** | **COVID-19 level β (95% CI)** | **February 2024 level β (95% CI)** |
| --- | --- | --- | --- |
| Composite, as originally specified | 71.3% | −5.1 (−7.3 to −3.0) | −8.7 (−11.8 to −5.6) |
| Composite, excluding transfer | 76.2% | −1.2 (−2.1 to −0.4) | −0.8 (−2.0 to 0.4), p=0.193 |
| KTAS 1–2 | 78.1% | −1.1 (−1.9 to −0.3) | −0.7 (−1.9 to 0.5), p=0.248 |
| ICU admission | 62.0% | −0.16 (−0.27 to −0.06) | −0.10 (−0.21 to 0.00), p=0.055 |
| In-hospital death | 62.3% | 0.01 (−0.03 to 0.05), p=0.731 | −0.04 (−0.08 to 0.00), p=0.062 |
| Inter-facility transfer-in | 69.1% | −4.0 (−5.8 to −2.2) | −7.8 (−10.5 to −5.2) |

All models use the primary specification: segmented linear regression on the monthly population-based rate with harmonic terms (sine and cosine, 12-month period) for seasonality and Newey-West heteroskedasticity- and autocorrelation-consistent standard errors (lag = 4). The components of the composite overlap, so these are component-specific analyses rather than an additive decomposition. The comparator for both composites is the non-resource group as originally defined (2023 Recovery Index 62.3%); visits qualifying solely through inter-facility transfer are therefore excluded from both groups in the transfer-excluded comparison.

**Facility-stratified results for the composite excluding inter-facility transfer:**

| **Facility tier** | **RI 2023** | **COVID-19 level β (95% CI)** | **February 2024 level β (95% CI)** |
| --- | --- | --- | --- |
| Regional emergency centers | 101.0% | −0.96 (−1.37 to −0.56) | −1.14 (−2.11 to −0.17), p=0.022 |
| Local emergency centers | 51.3% | −0.03 (−0.63 to 0.58), p=0.924 | +0.34 (0.03 to 0.65), p=0.031 |
| Other emergency facilities | 117.6% | −0.24 (−0.52 to 0.04), p=0.093 | −0.02 (−0.16 to 0.12), p=0.788 |

Rates use the national pediatric population as denominator and therefore represent population-level contact burden by facility tier, not facility-specific performance.

**Proportion of composite-qualifying visits that qualified solely through inter-facility transfer:**

| **Period** | **Composite (n)** | **Transfer-only (%)** |
| --- | --- | --- |
| Pre-COVID, 2018–2019 | 32,761 | 75.3% |
| Pandemic, Mar 2020–Dec 2022 | 35,010 | 78.0% |
| Recovery, 2023 | 11,680 | 73.6% |
| 2024 event, Feb–Dec 2024 | 7,324 | 66.2% |

Visits qualifying solely through inter-facility transfer are those that met no other composite criterion. Across the study period, 33,246 visits met the composite definition excluding transfer, against 125,889 for the composite as originally specified.

**eTable 7. Sensitivity of the primary model to the seasonality specification**

The primary model adjusts for seasonality using harmonic terms (sine and cosine, 12-month period). The table below refits the same model using eleven monthly indicator variables (January as reference), holding all other specifications constant.

| **Outcome** | **COVID-19 level β** |  | **February 2024 level β** |
| --- | --- | --- | --- |
|  | Harmonic | Monthly indicators | Harmonic / Monthly indicators |
| Overall injury rate | −183.3 | −187.3 | −157.9 / −159.8 |
| Regional emergency centers | −71.6 | −72.6 | −88.7 / −89.1 |
| Local emergency centers | −80.1 | −82.1 | −71.5 / −72.3 |
| Other emergency facilities | −31.7 | −32.7 | +2.4 / +1.6 (both ns) |
| Composite excluding transfer | −1.23 | −1.27 | −0.81 / −0.80 (both ns) |

Estimates are per 100,000 children per month. Absolute differences between the two seasonality specifications are small, and every direction of effect and statistical-significance conclusion is unchanged. ns = not statistically significant at the 5% level; for coefficients close to zero, small absolute differences correspond to larger relative differences without altering inference.
